# Complete Genome Sequence of *Thalassococcus* sp. Strain S3, a Marine *Roseobacter* Clade Member Capable of Degrading Carbazole

**DOI:** 10.1128/MRA.00231-19

**Published:** 2019-07-11

**Authors:** Felipe Vejarano, Chiho Suzuki-Minakuchi, Yoshiyuki Ohtsubo, Masataka Tsuda, Kazunori Okada, Hideaki Nojiri

**Affiliations:** a Biotechnology Research Center, The University of Tokyo, Tokyo, Japan; b Collaborative Research Institute for Innovative Microbiology, The University of Tokyo, Tokyo, Japan; c Graduate School of Life Sciences, Tohoku University, Sendai, Japan; University of Southern California

## Abstract

We determined the complete genome sequence of Thalassococcus sp. strain S3, a marine carbazole degrader isolated from Tokyo Bay in Japan that carries genes for aerobic anoxygenic phototrophy. Strain S3 has a 4.7-Mb chromosome that harbors the carbazole-degradative gene cluster and three (96-, 63-, and 46-kb) plasmids.

## ANNOUNCEMENT

Thalassococcus sp. strain S3 is one of several novel bacterial isolates from Tokyo Bay, Japan (1, 2), capable of degrading the mutagenic heteroaromatic carbazole (3, 4). Strain S3 belongs to a scarcely described genus (5, 6) of the marine Roseobacter clade, a ubiquitous group of alphaproteobacteria with several strains capable of aerobic anoxygenic photosynthesis (AAP) (7, 8). Some roseobacters can degrade monoaromatics through the beta-ketoadipate pathway (9, 10), and although there is one known Thalassococcus strain capable of phthalate degradation through this pathway, only enzymes for the degradation of intermediates are known (11). Similarly, available *Thalassococcus* genome sequences (GenBank accession number NZ_CP027665 and BioProject numbers PRJNA303447 and PRJNA481588) reveal beta-ketoadipate pathway genes, but studies on their degradation capabilities are absent. Here, we report the complete genome sequence of *Thalassococcus* sp. strain S3, a novel carbazole-degrading roseobacter that carries carbazole and intermediate degradation pathway genes and harbors an AAP plasmid.

**FIG 1:**
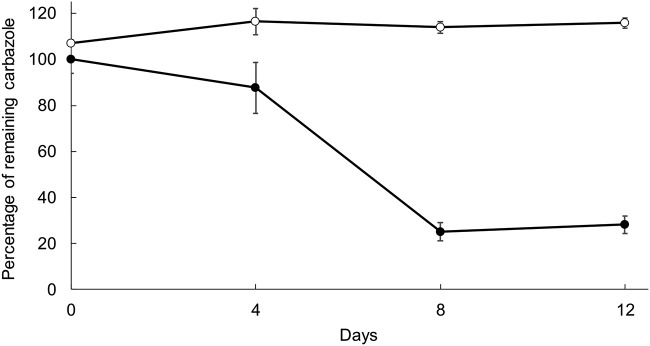
Carbazole degradation kinetics by *Thalassococcus* sp. strain S3. S3 cells precultured in CAR-SEA medium (2) containing 0.05% (wt/vol) carbazole were added to test tubes of fresh medium and incubated for 12 days at 30°C with shaking (300 strokes/minute) (filled circles). Every 4 days, the amount of remaining carbazole was measured. For every measurement, the entire culture was acidified, and carbazole was extracted with ethyl acetate. High-performance liquid chromatography (HPLC) analysis was performed on a Hitachi L-2000 platform using a Pegasil-B octadecyl-silica (ODS) column (4.6 by 250 mm; Senshu Kagaku) with water-acetonitrile as the mobile phase. Dibenzo-*p*-dioxin at a final concentration of 0.05% (wt/vol) was used as an internal standard. The remaining carbazole percentage was calculated using the ratio of carbazole signal to internal standard signal relative to that of a standard solution of known concentration of both compounds. The abiotic control consists of 0.05% (wt/vol) carbazole-containing CAR-SEA medium without inoculum (empty circles). Data are expressed as means ± standard deviation of the results from technical triplicates.

Enrichment and isolation of strain S3 were done using carbazole-containing, filter-sterilized seawater (CAR-SEA) medium, as previously described (2), while confirmation of carbazole degradation ability was done in CAR-SEA medium containing 0.05% (wt/vol) carbazole (Fig. 1). For genome sequencing, cells were grown in Marine broth 2216 (BD) for 3 days at 30°C with shaking (300 strokes/minute), and DNA was obtained as previously reported (2). The 6-kbp Nextera mate pair (MP) and 870-bp TruSeq PCR-free paired-end (PE) libraries (Illumina) were prepared following the manufacturer’s instructions and sequenced on a V3 chemistry, 600-cycle cartridge with a MiSeq platform (Illumina). Ten initial scaffolds were obtained using Newbler 2.8 (Roche; overlapMinMatchIdentity = 98; allContigThresh = 0) with 1 million PE reads (234 Mb) and 0.54 million MP reads (98 Mb) that were quality filtered using ShortReadManager 0.995 (threshold values, read score = 30; read length = 21; 21-mer frequency = 2) (12). Three of these scaffolds corresponded to contigs without gaps of complete plasmid sequences, identified as such upon detection of typical plasmid partition and replication genetic elements (putative DnaA boxes and *parA*, *parB*, *repA,* and *repB* homologs). Gaps between the remaining scaffolding contigs were identified as repeats induced by GenoFinisher 2.0 (default settings), and they were manually closed *in silico* with AceFileViewer 1.4 (default settings) (12), completing the sequence assembly of the chromosome. AceFileViewer also served to identify reads spanning contig boundaries, confirming the circularity of each assembly. Finally, FinishChecker 2.0 (default settings) was used with the MP data to find and correct assembly errors by detecting 21-mers that are absent in the reads but present in the finished sequences and by searching for abnormal distance distributions of mapped MP reads (13).

Gene annotation was done using PGAP (14) and MiGAP (15), and the annotated sequences were checked for inconsistencies in the identification of start/stop codons between both annotation pipelines using the CompareSequences tool of GenomeMatcher 2.203 (default settings with the bacterial RefSeq database, release 82) (16). Upon verification, these annotation results were merged into one curated annotation file.

*Thalassococcus* sp. S3 has one 4,707,308-bp chromosome (64× coverage) and three circular plasmids, pS3A, pS3B, and pS3C (95,560 bp, 63,231 bp, and 45,797 bp with 44×, 47×, and 48× coverage, respectively). The G+C contents of the chromosome, pS3A, pS3B, and pS3C are 60.9%, 62.8%, 62.5%, and 62.6%, respectively. A total of 4,791 coding sequences, 43 tRNA genes, and three rRNA genes were annotated. A 50-kb AAP gene cluster (17) was found on plasmid pS3A, while carbazole-degradative *car* genes were found on the chromosome. Interestingly, the 10.2-kb *car* gene cluster, which has the configuration *carBEFDAcCAa*(ORF:CFI11_19430)*carAd* (18), includes the gene for an extradiol dioxygenase structurally related to a 2,3-dihydroxybiphenyl-1,2-dioxygenase (CarB) (19) instead of the 2′-aminobiphenyl-2,3-diol 1,2-dioxygenase of other carbazole degraders (3, 20, 21). Last, an anthraniloyl-coenzyme A gene cluster for the degradation of the intermediate anthranilate (22) was found in place of genes for the anthranilate dioxygenase that leads to the beta-ketoadipate pathway.

### Data availability.

The genome sequence of *Thalassococcus* sp. strain S3 has been deposited in DDBJ/ENA/GenBank under the accession numbers CP022303, CP022304, CP022305, and CP022306 for the chromosome and plasmids pS3A, pS3B, and pS3C, respectively. The details of the software parameters and description of bioinformatics tools for the assembly of each replicon are available in the comments section of the accession number entries. Raw sequencing data have been deposited in the SRA under accession numbers SRX5129140 (PE data) and SRX5129141 (MP data).
